# Prognostic Potential of Nectin Expressions in Colorectal Cancer: An Exploratory Study

**DOI:** 10.3390/ijms242115900

**Published:** 2023-11-02

**Authors:** Jakub Kobecki, Paweł Gajdzis, Grzegorz Mazur, Mariusz Chabowski

**Affiliations:** 1 Department of Surgery, 4th Military Teaching Hospital, 5 Weigla Street, 50-981 Wroclaw, Poland; jakubkobecki@gmail.com; 2 Division of Anaesthesiological and Surgical Nursing, Department of Nursing and Obstetrics, Faculty of Health Science, Wroclaw Medical University, 5 Bartla Street, 51-618 Wroclaw, Poland; 3 Department of Pathomorphology, 4th Military Teaching Hospital, 5 Weigla Street, 50-981 Wroclaw, Poland; pgajdzis@protonmail.com; 4 Department of Clinical Pathology, Wroclaw Medical University, 213 Borowska Street, 50-556 Wroclaw, Poland; 5 Department of Internal Medicine, Occupational Diseases, Hypertension and Clinical Oncology, Wroclaw Medical University, 213 Borowska Street, 50-556 Wroclaw, Poland; grzegorz.mazur@umw.edu.pl; 6 Department of Clinical Surgical Sciences, Faculty of Medicine, Wroclaw University of Science and Technology, 50-556 Wroclaw, Poland

**Keywords:** colorectal cancer, adhesion molecules, nectin, prognostic factors

## Abstract

Colorectal cancer (CRC) is a pressing global health challenge, with an estimated 1.9 million new cases in 2020. Ranking as the third most diagnosed cancer globally, CRC accounts for nearly 930,000 cancer-related deaths annually. Nectins, immunoglobulin-like adhesion molecules, are pivotal in intercellular adhesion formation and cellular function regulation. Altered nectin expression patterns have been identified in various cancers. However, the intricacies of their role in cancer development and progression remain underexplored. This study aimed to evaluate the expression of specific nectins in CRC tumors, explore their association with clinicopathological factors, and ascertain their potential as prognostic indicators for CRC patients post-resection. We retrospectively analyzed the medical records of 92 CRC patients who underwent surgical treatment between 2013 and 2014. Tumor specimens were re-evaluated to determine nectin expression using immunohistochemistry. The study identified heterogeneous expressions of nectin-2, -3, and -4 in 58%, 62.6%, and 87.9% of specimens, respectively. Elevated nectin-4 expression correlated with worse 5-year and overall survival rates, presenting a negative prognostic value (HR = 4, 95% CI: 2.4–6.8, *p* < 0.001). Conversely, reduced nectin-3 expression was linked to poorer CRC prognosis (HR = 0.54; 95% CI: 0.31–0.96; *p* = 0.036). Nectin-4 expression positively correlated with elevated carcinoembryonic antigen (CEA) levels and advanced disease stages. In contrast, nectin-3 expression negatively correlated with CEA levels, tumor size, presence of distant metastases, and disease stage. Notably, tumors in the right colon were statistically more likely to express nectin-2 compared to those in the left. This study underscores the potential prognostic significance of nectins in CRC. The high prevalence of nectin-4-expressing cells offers promising avenues for further evaluation in targeted therapeutic interventions with already available agents such as PADCEV.

## 1. Introduction

Colorectal cancer (CRC) remains a major global health concern, ranking as the third most common cancer with nearly 2 million new cases each year, according to the WHO [1]. It is the second biggest cause of cancer-related mortality, responsible for 935,000 deaths in 2020 [2]. Predictive models indicate a growing impact on healthcare systems and an alarming increase in incidence among adults under 50 [3,4]. Treatment costs are high, creating economic burdens for both developed and low-income countries. The situation has been further exacerbated by the COVID-19 pandemic, which led to delays in diagnosis and treatment [5]. Despite advancements in understanding CRC, 5-year survival rates have not seen significant improvement [6]. CRC is a molecularly and anatomically heterogeneous disease that evolves through a multi-step process involving the accumulation of various genetic or epigenetic mutations [7]. The complex nature of CRC, with variations in patient outcomes even within the same stage, highlights the need for continued research into prognostic and possibly predictive factors. Molecular biomarkers, such as microsatellite instability (MSI), CpG island methylator phenotype (CIMP), chromosomal instability (CIN), and mutations in genes like KRAS and BRAF, have emerged as significant in evaluating the prognosis of CRC [8]. Additionally, microRNAs (miRNAs), short non-coding RNAs, have emerged as potential diagnostic and prognostic biomarkers due to their role in various biological processes and immune responses [7]. One focus is on cell adhesion molecules, which are being explored for their diagnostic and therapeutic potential in CRC [9,10].

Nectins are a group of type I transmembrane proteins that belong to the immunoglobulin superfamily and function as cell adhesion molecules (CAMs). They are closely related to but distinct from nectin-like molecules (necl) [11]. Nectins have four primary members (nectin-1 to -4). Nectins-1 to -3 are usually found in adult tissues, while nectin-4 is mainly seen in embryonic and placental tissues. Nectins play a role in cell adhesion by forming both same-type (homophilic) and different-type (heterophilic) bonds. These interactions are crucial for various developmental processes and for maintaining tissue stability. Nectins can interact with each other (e.g., nectin-4 with nectin-1) as well as with necl family members. Through these interactions, nectins participate in various biological processes, including immune responses, cell growth, and migration [12,13]. According to the available scientific literature, cancer diseases may be accompanied by significant variability in nectin expression on the surface of tumor cells [14]. The significance of this variability and potential clinical use in CRC has not yet been well researched. The fact that in 2019, the American FDA (Food and Drug Administration) approved the use of a cancer drug conjugate with a monoclonal antibody directed against nectin-4 in the treatment of metastatic urothelial cancer opens up possibilities for its potential application in different neoplasms [15]. In light of the above, expanding knowledge about nectins’ expression and their use as cancer biomarkers, including CRC, is becoming an important subject of research.

Surgical treatment remains fundamental for CRC management, especially in the early stages. Although the fundamental goals of surgery have not changed significantly, there have been advances in surgical techniques over the past three decades. The field has steadily moved towards minimally invasive approaches, such as minimal access or keyhole surgery, which improve patient outcomes [16,17]. The future of colorectal cancer treatment likely lies in the intersection of advanced statistical tools and individualized care. Techniques such as the Consensus Molecular Subtypes in Colorectal Cancer (CMS) offer a robust framework for prognostic and predictive analysis, allowing clinicians to classify patients into more clinically meaningful categories based on various biomarkers [18,19]. The aim of this paper is to assess the expression of selected nectins and their prognostic significance in CRC.

## 2. Results

**Figure 1:**
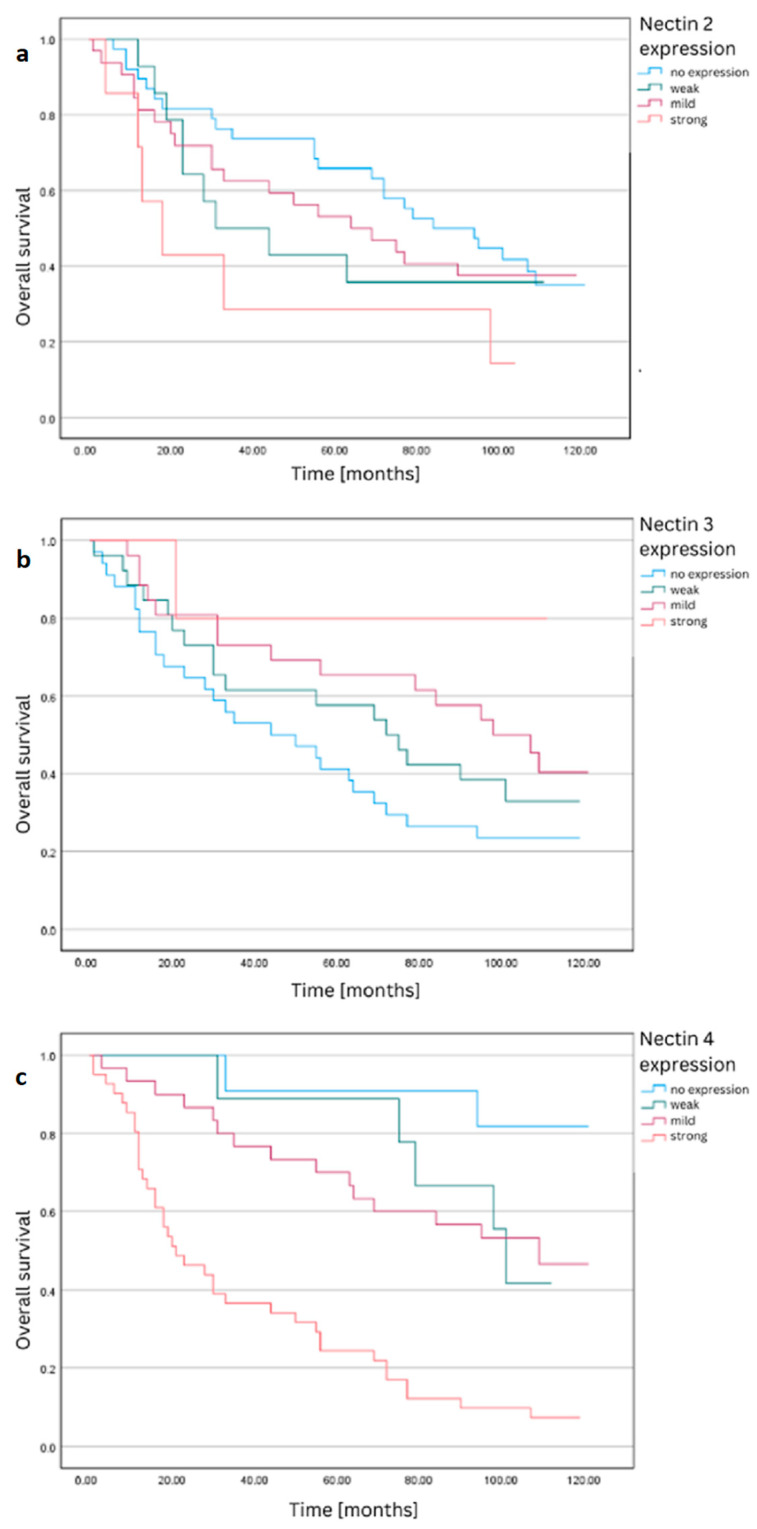
Overall survival functions for the (**a**) nectin-2, (**b**) -3, and (**c**) -4 expression based on IRS classification.

To investigate the differences in 5-year survival, we conducted functions based on the negative, low, medium, or high expression of nectins (-2, -3, and -4) using the IRS (immunoreactivity scoring system), Kaplan–Meier survival analyses (Table 1). The log-rank test was statistically significant only for nectin-4 expression, indicating significant differences in the survival function. Patients with a high nectin-4 expression had the shortest average survival and the highest mortality. The survival functions are shown in Figure 1.

**Figure 2:**
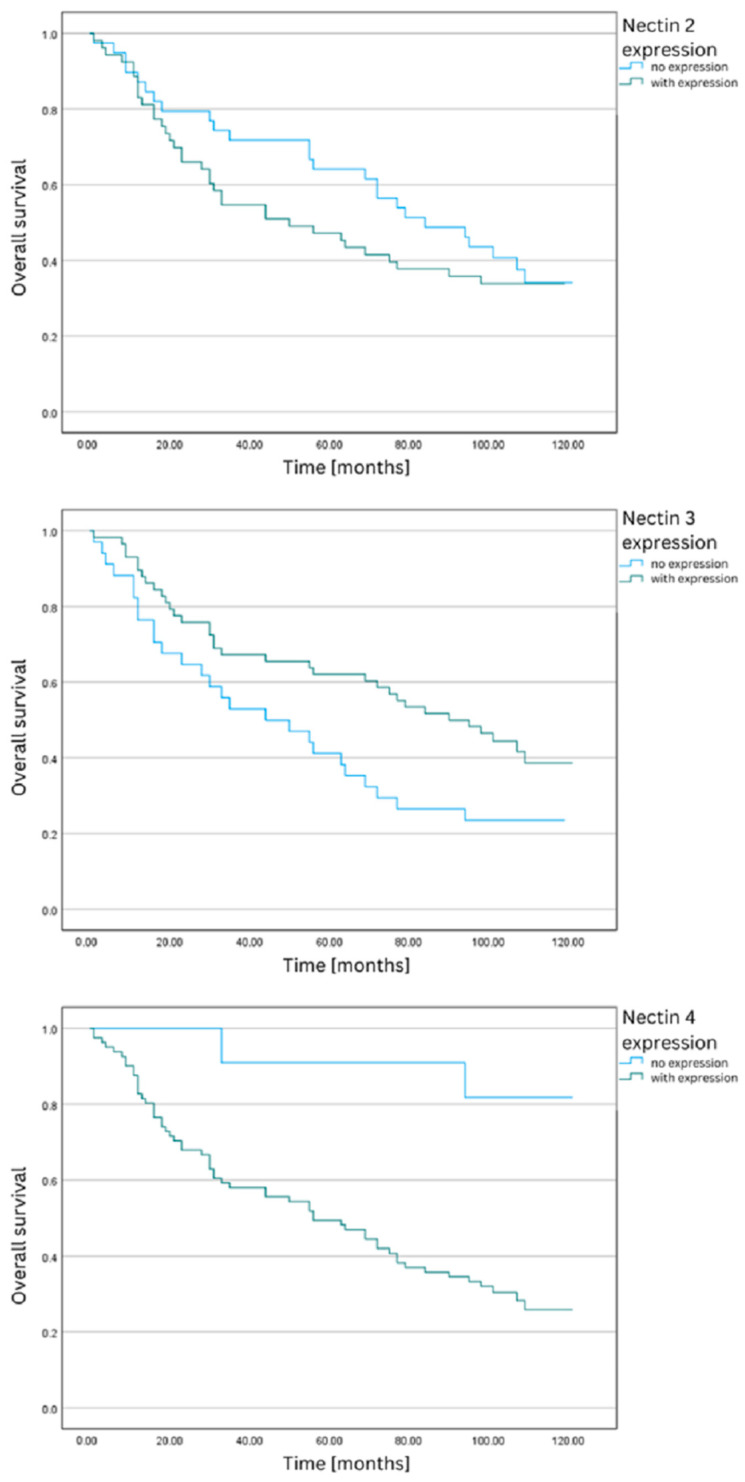
Overall survival functions for nectin-2, -3, and -4 based on alternative IRS classification (0–1) vs. (2–12).

Based on prior findings for nectin-3, an additional survival analysis was conducted using an alternative IRS classification. Patients were divided into groups with no expression (IRS 0–1) and positive expression (previously categorized as weak, mild, and strong, IRS 2–12). Table 2 displays the analyzed frequencies, average survival durations, standard deviations, 95% confidence intervals, and log-rank test results. The results were statistically significant for nectin-3 and -4, indicating significant differences in the survival functions. Patients with nectin-4 expression and without nectin-3 expression had the shortest average survival time and highest mortality rates. The survival curves are shown in Figure 2.

We also analyzed the expression levels of nectin-2, -3, and -4, utilizing the previously established stratification methods, to assess the risk of mortality during the entire observation period. This analysis was performed using the Cox proportional hazards model. The findings are presented in the form of hazard ratios (HR) accompanied by 95% confidence intervals (Table 3). In line with the earlier presented results, it is noteworthy that the observed elevated risk of mortality among patients exhibiting higher nectin-2 expression did not attain statistical significance.

To investigate the relationships between the expression of nectin-2, -3, and -4 (IRS classification) and other variables measured, a correlation analysis was conducted using Spearman’s rho and Pearson’s r coefficients. Based on the results presented in Table 3, it was found that the expression of nectin-3 was negatively and weakly associated with the preoperative serum CEA concentration, tumor diameter, and CRC stage. This means that as the concentration of CEA increased, and as the diameter of the tumor measured in cm and the CRC stage of advancement increased, the expression of nectin-3 decreased. In the case of nectin-4, it was found that its expression was positively and moderately associated with the preoperative serum CEA concentration and CRC stage of advancement. This means that as the concentration of CEA and the CRC stage of advancement increased, the observed expression of nectin-4 increased. The correlation of nectin-4 expression with the T feature was on the borderline of statistical significance.

To verify the relationships between the expression or lack of expression of nectin-2, -3, and -4 and various factors such as tumor location (right or left half of the colon and colon vs. rectum), the degree of histological malignancy, gender, habits, as well as T, N, and M stages, a series of analyses were conducted using the chi-square test for independence. Results with statistical significance are presented in Table 4. A significant association was observed between the expression of nectin-2 and tumor location; upon analyzing the adjusted standardized residuals, it was found that the expression of nectin-2 was associated with tumors located in the right half of the colon (Table 5).

## 3. Discussion

Based on the available literature, it is not possible to unequivocally define universal criteria for the tissue expression of nectins via immunohistochemistry (IHC). Depending on the tissues studied and the methodology selected, authors often arbitrarily adopt assumptions concerning baseline, appropriate, physiological, or pathological expression levels. Hence, in our results, one can observe various approaches to analyzing the degree of expression. The absence of standardized tools for evaluating nectin expression in cancer cells makes scientific discourse and the comparison of results between the relatively few published papers even more challenging. For better clarity, the discussion that follows is divided into subsections dedicated to each nectin evaluated.

### 3.1. Nectin-2

Depending on the source, nectin-2 is cited as either ubiquitously present or virtually absent in healthy tissues, including the colon. A consensus among various authors suggests the presence of nectin-2 expression in tissues of the immune system [20,21,22,23]. Numerous publications indicate overexpression of nectin-2 in epithelial-origin tumors and adenocarcinomas, including colorectal cancer [12,22,24,25,26,27,28,29]. 

The literature offers a scant number of publications addressing nectin-2 expression in CRC. Zhang et al. conducted a study on nectin-2 expression in CRC cells and its role in NK cell-dependent cytotoxicity. They observed nectin-2 expression in 52.4% of the specimens examined through immunohistochemistry and higher serum concentrations of nectin-2 in CRC patients compared to the control group. No correlation was found between nectin-2 expression and measured clinicopathological factors [30]. Karabulut et al. assessed serum concentrations of nectin-2 in 140 CRC patients. The levels of nectin-2 had both diagnostic and prognostic significance for patients at stages I to III; higher serum levels were statistically associated with shorter progression-free survival [31]. The study did not find a relationship between serum nectin-2 levels and overall survival in the patient cohort or their response to adjuvant therapy. The authors suggest that the difference in baseline serum nectin-2 levels between CRC patients and the healthy control group could be due to a higher expression of this molecule within the tumor, although this was not investigated. Similar findings were presented on lung cancer patients [32]. In our study cohort, established by the criteria we adopted, nectin-2 was present in 57.6% (>1 IRS score) of the examined specimens (Table 1 and Table 2). Compared to the study by Zhang et al., this is a very similar result [33]. However, any comparison of the results is fraught with significant distortion due to the lack of precisely defined methodology for evaluating expression. Only the papers that inspired Zhang’s team can provide information about possible compatibility with our classification—both point systems require cases to meet a >50% cell expression criterion or at least a medium intensity of staining to qualify as positive. An attempt to compare our study group with cohorts of patients with ovarian, breast, and pancreatic neuroendocrine tumors is further complicated by even greater methodological and especially biological differences [20,21]. Future studies should aim to create a comprehensible system for immunohistochemical assessment of nectin-2 expression based on healthy and neoplastic tissues. It would be insightful to conduct research comparing the expression results of the same population using different evaluation systems. Existing intriguing reports on the significance of nectin-2 in neoplastic disease encourage further research in this area. Promising results in measuring serum nectin-2 concentrations indicate another interesting avenue for future studies. Does serum nectin-2 concentration correlate with its tumor expression in CRC patients? Does a high preoperative serum concentration of nectin-2 decrease depending on the radicality of the operation? These are questions that remain unanswered in the existing literature. 

Based on the analysis conducted within this study, there is significant heterogeneity in the expression of nectin-2 in CRC tissues. Despite a noticeable trend in 5-year and overall survival time (greater average months survived) in the group characterized by the absence of nectin-2 expression, no statistically significant difference was obtained between these groups (Table 1, Table 2 and Table 3, Figure 1 and Figure 2). Limitations to our analysis certainly include the sample size and the evaluation of individual tumor specimens. Tumor heterogeneity and inter-individual differences could be reflected in varied protein expression within the tumor.

A surprising result was the correlation between nectin-2 expression and tumor location. The presence of nectin-2 expression positively correlated with the proximal CRC location. According to the existing literature, tumors located in the right half of the colon are associated with a worse prognosis [34,35,36]. In this study, no statistically significant difference in 5-year and overall survival was observed between patient groups with tumors in the right and left half of the colon. This could also have been influenced by the small sample size of the study. Based on current knowledge concerning the observed molecular profile differences between right and left colon cancers, it appears interesting that nectin-2 may be associated with tumor sidedness [37]. To our knowledge, no scientific work has been conducted to date that addresses the issue of nectin expression and tumor sidedness. 

### 3.2. Nectin-3

Nectin-3 is present in healthy, mature human tissues, including the gastrointestinal tract. It is found on the surface of T lymphocytes and plays a role in the organogenesis of organs such as the eye and cerebral cortex [12,23,25,38].

The studies cited above unequivocally indicate a relationship between nectin-3 expression and a tumor phenotype with a poorer prognosis. These studies focus on tumors of tissues that, under normal conditions, exhibit little or no expression of nectin-3. Elevated expression of nectin-3 has been observed in various types of cancers, including lung adenocarcinomas, ovarian, and nasopharyngeal cancers [39]. Physiologically, nectin-3 is present on the surface of cells in the mucous membrane of the colon and acts as an obligatory receptor for toxin B (TcdB) produced by C. difficile [40]. Studies have shown that its overexpression in some cancers, for example lung adenocarcinomas, was associated with poor prognosis [41]. 

We investigated the relationship between nectin-3 expression and patient survival in CRC patients. Our findings revealed a significant negative correlation between reduced nectin-3 expression and patient survival. Specifically, the Hazard Ratio (HR) associated with decreased nectin-3 expression was calculated to be 0.54 (95% CI: 0.31–0.96; *p* = 0.036). This result indicates that patients with reduced nectin-3 expression faced a significantly higher risk of mortality compared to those with higher nectin-3 expression.

These results are consistent with previous research in various cancer types, including pancreatic adenocarcinomas, neuroendocrine tumors, and breast cancer [28,42,43]. In those studies, reduced nectin-3 expression was associated with higher malignancy, poorer prognosis, and adverse clinical features such as larger tumor size, metastases, and shorter progression-free survival. The potential role of nectin-3 as a tumor suppressor molecule and its influence on cell junction formation have been highlighted in these studies. These findings underscore the potential clinical relevance of nectin-3 in risk stratification, and further research in this area may provide valuable insights into clinical implications.

What should be emphasized here once again are the difficulties associated with the lack of consensus on immunohistochemical study methodology among the authors of the available publications. Based on the descriptions of studies by Hirabayashi and Izumi et al., it can be concluded that the determining factor was solely the percentage of cells presenting the antigen, and, in both cases, with slight variations, samples with more than half of the cells that were “positive” were classified in groups with “high” or “positive” expression [42,43].

Correlation analysis demonstrated a relationship between nectin-3 expression, tumor size, and preoperative CEA levels. As observed in the study group, with an increase in tumor dimension and CEA levels, nectin-3 expression decreased (Table 3). A statistically significant correlation was also found between the stage of advancement and nectin-3 expression, whose decline was accompanied by higher stages of colorectal cancer (CRC) advancement. All these relationships can be explained by mechanisms related to more malignant tumor phenotypes with a worse prognosis. Both tumor size and preoperative CEA level prognostic factors in CRC [44,45,46,47,48,49,50].

We did not observe correlations between the degree of nectin-3 expression and the individual elements of the TNM system, as was the case in the aforementioned studies by Hirabayashi et al. on pancreatic neuroendocrine tumors. Different observations may arise from the different nature of the tumor. There are no available studies that would allow us to compare our results with patient groups suffering from CRC. 

The available literature on nectin-3 in neoplastic diseases is inconsistent and is very limited in the matter of CRC patients. The role of nectin-3 in processes related to cancer progression, such as invasion, metastasis formation, and prognosis, seems to vary depending on the histological type and origin of the tumor. Consideration should be given to expanding the scope of research on the significance of nectin-3 in CRC. A promising approach could be to compare the properties of cancer cells with induced nectin-3 overexpression and after loss of nectin-3 expression (e.g., using specific siRNAs). What accounts for such a varied relationship between nectin-3 expression and prognosis in other cancers? Would reconstitution of lost nectin-3 expression inhibit CRC development in a cellular model? Future studies on nectin-3 should provide answers to these questions.

### 3.3. Nectin-4

Nectin-4 is another member of the nectin family that has significant implications both in physiology and in various diseases. Although it is weakly expressed or nearly absent in healthy adult tissues, nectin-4 plays a crucial role during embryonic development, contributing to organogenesis [29,38,51]. Numerous reports indicate that nectin-4 is overexpressed in a wide array of cancers such as colorectal, gastric, esophageal, breast, ovarian, hepatocellular, non-small-cell lung, urothelial, papillary thyroid, and renal cancers [14,25,27,29,52]. This overexpression suggests that it may serve as a potential biomarker or therapeutic target in the treatment of these malignancies.

In the population studied, nectin-4 was the most commonly occurring among the selected nectins. Its expression was observed in as many as 87.91% of cases, based on the ImmunoReactive Score (IRS) classification. Compared to the existing literature, these are higher percentages than those reported by other researchers. A study conducted by Challita-Eid et al. assessed the expression of nectin-4 in 2394 selected cancer cases [15]. Considering the differences in the method of assessing nectin expression (H-score vs. IRS), it can be assumed that the cutoff point for detecting nectin-4 expression in both studies will be similar.

In the aforementioned study, the highest percentage of positive samples was found in the group of bladder cancer—83%, and the lowest in lung and esophageal cancers—55%. There were no cases of colorectal cancer (CRC) among the tumors studied. Considering these results, it should be noted that the percentage of tumors expressing nectin-4 in CRC is at least comparable to the tumors analyzed by Challita-Eid et al [15].

Moretto et al. used samples from patients diagnosed with metastatic CRC from the TRIBE2 study to investigate nectin-4 expression and its relationship with observed clinical and demographic characteristics [53]. The authors note the diversity of nectin-4 expression and emphasize that any targeted therapy should be preceded by testing for nectin-4 expression. The interpretation of the H-score result by the authors makes it difficult to compare the results with our study.

Significant differences in survival were demonstrated over both 5-year and overall periods, depending on the degree of nectin-4 expression (Table 1 and Table 2, Figure 1 and Figure 2). Notably, patients with higher nectin-4 expression in CRC cells exhibited statistically worse prognosis in all analyzed timeframes. Survival function graphs vividly illustrate the distinctions between the absence of expression and weak, moderate, and high expression, as categorized by the IRS classification. Additionally, a statistical difference was observed in the survival rates when dividing patients based solely on the percentage of cells expressing nectin-4. Although the function graphs, in this case, may not reveal differences as pronounced as those based on the IRS classification, our analysis indicates that nectin-4 expression is significantly associated with a poorer prognosis in the assessed time periods. Of particular note is the Hazard Ratio (HR) for nectin-4, which stands at an alarming 4, further underscoring its profound prognostic value for patients with CRC. 

Using nectin-4 as an illustrative example, our choice of the IRS classification appears to be apt for stratifying groups based on progressively increasing nectin-4 expression, revealing statistically significant differences in predicted survival. Our findings align with those of a recently published meta-analysis investigating the prognostic implications of nectin-4 across various cancer types [54]. Regrettably, within the analyzed literature, no studies specific to colorectal cancer patients were identified.

This lack highlights the importance of our study, as it contributes valuable insights into the prognostic relevance of nectin-4 within the context of colorectal cancer—a topic that warrants further exploration.

Given the high percentage of colorectal cancer (CRC) cells observed in our study group showing nectin-4 expression, it seems reasonable to consider a clinical trial involving patients with colorectal cancer. Based on the experiences of other researchers, it should be noted that tumor heterogeneity and inter-individual differences may be reflected in the varied expression of proteins, resulting in variable patterns of immunohistochemical staining. Changes in expression may also occur between primary tumors and metastatic tumors, which could be critical for the efficacy of ADC-based therapy. These factors should be accounted for in the design and interpretation of clinical trials.

Our study possesses several notable limitations that warrant discussion. First, the sample size, though carefully selected, remains a limitation in terms of statistical power and generalizability. Second, tumor heterogeneity is inherent in colorectal cancer, which may introduce variability in our findings. Additionally, the heterogeneity in nectin expression across patients adds complexity to the analysis. Third, our study design is retrospective, and as such, it is subject to the limitations associated with retrospective data collection, including the potential for bias and incomplete data. Finally, we acknowledge the use of polyclonal antibodies in our immunohistochemistry (IHC) methods, which can introduce variability in staining and may not account for all isoforms or epitopes. These limitations should be considered when interpreting our results, and future research should aim to address these challenges to advance our understanding of colorectal cancer.

There are still many issues related to nectin-4 and its significance in CRC that require further clarification. The standardization of the method of detecting nectin-4 expression, the assessment of heterogeneity of expression within the tumor, and secondary lesions are just a few of them.

## 4. Materials and Methods

### 4.1. Study Design

The study is retrospective, non-interventional, and based on re-evaluation of histopathological specimens collected during therapeutic surgical procedures. The research did not pose any risk to participants. The study group consists of colorectal cancer patients who underwent surgery at the Surgical Clinic of the 4th Military Teaching Hospital in Wroclaw between 2013 and 2014.

### 4.2. Patient Selection and Data Collection

Medical records of patients with diagnoses ICD-10: C18, C19, or C20 were analyzed. Due to different etiology, prognosis, and treatment, patients with anus and anal canal cancers (ICD-10 C21) were not included in the study. After preliminary analysis and verification, 106 patients were included in the further study. We obtained paraffin blocks, and histopathological slides of all included patients from the archive of the Department of Pathomorphology of the 4th Military Teaching Hospital. Technical difficulties encountered during the immunohistochemical process meant that some of them were not suitable for reliable evaluation. Ultimately, 92 patients were qualified for the study. Subsequently, the patients were followed up, taking into account the dates and causes of deaths, if they occurred. For the total survival time, the end of observation was set for 25 February 2023, when the final report of the Civil Registry Office in Wroclaw confirming the status of patients in the study was received.

The study group included 37 women (40.22%) and 55 men (59.78%) aged between 48 and 90 years. (Table 6) Only 1 out of 92 patients (1.08%) was under 50 years of age. The average age of a patient at the time of surgery was 67.93 years. A total of 61 patients (66.3%) had cancers of the left side of the colon and 31 of the right side of the colon (33.7%). The characteristics of the study group are reflected in the general structure of morbidity in terms of sex, age, and tumor location among Polish patients at that time. All the cases included in the study had been diagnosed histopathologically as adenocarcinomas of the colon.

### 4.3. IHC Analysis

For the evaluation of nectin-2, nectin-3, and nectin-4 protein expression, the immunohistochemical method was employed. Paraffin blocks for immunohistochemical tests were selected based on histopathological specimens stained with the HE method. For each case, one paraffin block was chosen. From the selected blocks, specimens were prepared for immunohistochemical staining against antibodies: nectin-2 (polyclonal antibody with catalog number PA5-82470, applied dilution 1:200), nectin-3 (polyclonal antibody with catalog number PA5-82410, applied dilution 1:75), and nectin-4 (polyclonal antibody with catalog number PA5-98636, applied dilution 1:250) (Invitrogen, Carlsbad, CA, USA). Immunohistochemical stains were performed using an Autostainer Link 48 (DAKO) staining machine. The Envision FLEX+ (DAKO) visualization kit and DAB (3,3′-diaminobenzidine) as the chromogen were used to visualize the reactions. Following the manufacturer’s antibody instructions, external controls were applied: for nectin-2 and-3—placental tissue and for nectin-4—pancreatic adenocarcinoma.

To evaluate the expression of nectins in histopathological specimens, the IRS (immunoreactive score) system was used, which comprises the product of the point equivalent of the percentage of cells presenting the antigen and the point equivalent of the staining intensity of the specimen (Table 7) [55,56,57,58].

The result was interpreted according to the criteria presented in Table 6. In the statistical analysis, a division was also used into patients in whom expression was not detected in the specimens (IRS 0–1 pts.) and patients in whom expression was detected in the specimens (IRS 2–12 pts.).

**Figure 3:**
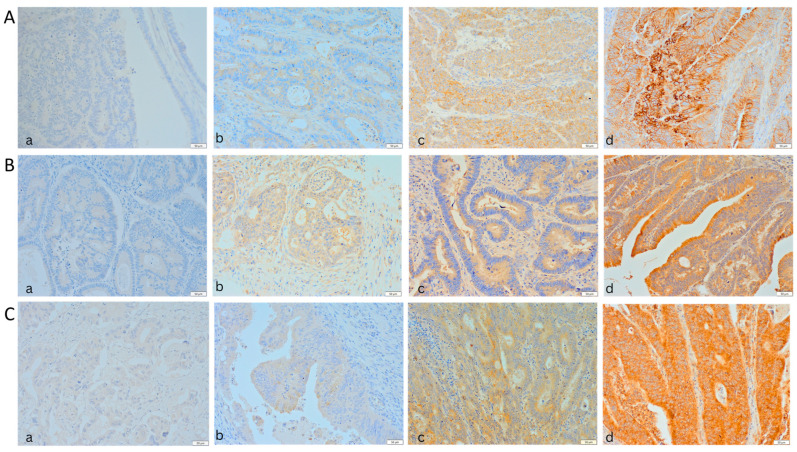
IRS Classification in sample specimens of the study group. Nectin-2 (**A**) strong (**a**), mild (**b**), weak (**c**), and without (**d**) expression. Nectin-3 (**B**) strong (**a**), mild (**b**), weak (**c**), and without (**d**) expression. Nectin-4 (**C**) strong (**a**), mild (**b**), weak (**c**), and without (**d**) expression. (scale: 50 μm).

The immunohistochemical reactions were jointly evaluated by Dr Paweł Gajdzis and Dr Jakub Kobecki. A light microscope (Olympus BX46) with a digital camera (Olympus SC180) and an additional external screen were used, allowing for simultaneous viewing of the specimens (Olympus, Tokyo, Japan). The evaluators individually assigned points in the IRS classification. In cases where the scores were inconsistent, all discrepancies were discussed, and a common IRS classification result was agreed on by both evaluators. In the specimens evaluated, nectin-2 mainly showed membrane expression and partially membrane-cytoplasmic expression. The observed expressions of nectin-3 and -4 were primarily cytoplasmic and partly membrane-cytoplasmic. Selected examples of specific degrees of expression according to the IRS classification are illustrated in Figure 3.

### 4.4. Statistical Analysis

Statistical analysis was conducted using SPSS Statistica version 28.0.1. Patient survival time, based on the degree of nectin expression in histopathological specimens, was assessed using Kaplan–Meier survival estimations and log-rank tests. Additionally, the Cox proportional hazards model was employed to determine hazard ratios (HR) for nectin expression levels, providing insights into their impact on patient survival. To compare differences between quantitative and ordinal variables, we initially used the *t*-test for independent samples. Due to non-normality and substantial group size differences, we complemented these analyses with non-parametric Mann–Whitney tests. Normality checks were performed using the Shapiro–Wilk test. A significance level of 0.05 was adopted for all statistical tests, with *p*-values below 0.05 considered statistically significant. It is important to note that our collaboration with an experienced medical statistician ensured the reliability of our research and the appropriate selection of statistical methods.

## 5. Conclusions

Colorectal cancer (CRC) continues to be a significant global health challenge, with its incidence and mortality rates remaining a concern. This study delved into the role of nectins, specifically nectin-2, -3, and -4, in their potential role as prognostic markers. Our findings indicate:

1. A heterogeneous expression of nectins was observed in the tumor specimens, with nectin-4 showing the highest expression.
2. A higher expression of nectin-4 was associated with poorer 5-year and overall survival rates, suggesting its potential as a negative prognostic marker.
3. Conversely, the loss of nectin-3 expression was linked to a worse prognosis, highlighting its importance in CRC progression.
4. Nectin-4’s expression showed a positive correlation with Carcinoembryonic Antigen (CEA) levels and advanced disease stages, while nectin-3’s expression was inversely related to these factors.
5. The potential of nectins, especially nectin-4, as targets for therapeutic interventions is promising, given their significant expression in most CRC cells.

The study underscores the importance of nectins in CRC progression and their potential as prognostic markers. Further research is required to standardize the evaluation of nectin expression and to explore their therapeutic potential in CRC management.

## Figures and Tables

**Table 1:** Hazard Ratios (HR) and associated results for different nectin expressions.

Nectin and Expression Method	Covariate(B)	HR (95% CI)	Wald Test	*p*-Value
Nectin-2 Expression According to IRS Classification.	0.17	1.2 (0.9–1.6)	1.5	0.22
Nectin-3 expression according to alternative IRS Classification (0–1) vs. (2–12).	−0.61	0.54 (0.31–0.96)	4.4	0.036
Nectin-3 Expression According to IRS Classification.	−0.16	0.29 (0.3–0.76)	2.1	0.28
Nectin-4 Expression According to IRS Classification.	1.4	4 (2.4–6.8)	27	<0.001

**Table 2:** Five-year survival analysis for nectin-2, -3, and -4 expression, according to IRS classification.

							95% CI		
Nectin	Expression	No. of Patients	No. of Deaths	5-Year Survival	M	SE	LLCI	ULCI	χ^2^
	No expression	39	14	64.10%	47.44	3.25	41.07	53.80	5.98
	Weak expression	14	8	42.86%	39.29	5.07	29.35	49.22	
2	Mild expression	32	15	53.13%	42.69	3.82	35.21	50.17	
	Strong expression	7	5	28.57%	28.71	8.06	12.93	44.50	
	No expression	34	20	41.18%	38.35	3.82	30.86	45.85	5.17
	Weak expression	26	11	57.69%	43.96	4.15	35.82	52.10	
3	Mild expression	26	9	65.38%	47.73	3.73	40.42	55.04	
	Strong expression	5	1	80.00%	52.20	6.98	38.53	65.87	
	No expression	11	1	90.91%	57.55	2.34	52.96	62.13	31.90 ***
	Weak expression	9	1	88.89%	56.78	3.04	50.82	62.73	
4	Mild expression	30	9	70.00%	50.17	3.16	43.98	56.35	
	Strong expression	41	31	24.39%	30.68	3.37	24.08	37.29	

M—Mean; SD—Standard Deviation; LL and UL—Lower and Upper Limit of the 95% Confidence Interval; χ^2^—Chi-Squared Test Result. ***—*p* < 0.001.

**Table 3:** Five-year survival analysis for nectin-2, -3, -4 expression, according to alternative IRS classification (0–1) vs. (2–12).

							95% CI		
Nectin	Expression	No. of Patients	No. of Deaths	5-Year Survival	M	SE	LLCI	ULCI	χ^2^
2	No expression	39	14	64.10%	47.44	3.25	41.07	53.80	2.56
	With expression	53	28	47.17%	39.94	2.94	34.18	45.70	
3	No expression	34	20	41.18%	38.35	3.82	30.86	45.85	3.98 *
	With expression	58	22	62.07%	45.91	2.64	40.74	51.09	
4	No expression	11	1	90.91%	57.55	2.34	52.96	62.13	5.56 *
	With expression	81	41	49.38%	41.16	2.42	36.43	45.90	

M—Mean; SE—Standard Deviation; LL and UL—Lower and Upper Limit of the 95% Confidence Interval; χ^2^—Chi-Squared Test Result. *—*p* < 0.050.

**Table 4:** Correlation coefficients of the expression of nectin-2, -3, and -4 and other variables were measured.

		Nectin-2	Nectin-3	Nectin-4
CEA	ρ-value	0.13	−0.33	0.45
	*p*-value	0.233	0.003	<0.001
Tumor size	ρ-value	−0.08	−0.30	0.09
	*p*-value	0.478	0.003	0.386
Stage	ρ-value	0.07	−0.28	0.43
	*p*-value	0.485	0.007	<0.001
T	ρ-value	−0.02	−0.09	0.20
	*p*-value	0.867	0.382	0.053

**Table 5:** Cross-table for the expression of nectin-2 and tumor location.

			Nectin-2 Expression		χ^2^
			No Expression	With Expression	
Tumor location	Left side	*n*	32	29	7.51 **
		%	34.8%	31.5%	
			(2.7)	(−2.7)	
	Right side	*n*	7	24	
		%	7.6%	26.1%	
			(−2.7)	(2.7)	

Note: Adjusted standardized residuals are provided in parentheses. **—*p* < 0.010.

**Table 6:** Descriptive statistics.

	M	SD	Me
Age	67.93	10.21	66.00
BMI	25.48	4.45	24.59
		*n*	%
Gender	Female	37	40.22
	Male	55	59.78
CRC location	Left side	61	66.30
	Right side	31	33.70
Smoking	No	56	60.87
	Yes	36	39.13
Alcohol abuse	No	86	93.48
	Yes	6	6.52
Type of surgery	- Abdominoperineal resection of the rectum	2	2.17
	- Anterior resection of the rectum	43	46.74
	- Left hemicolectomy	11	11.96
	- Right hemicolectomy	29	31.52
	- Sigmoidectomy	4	4.35
	- Transverse colectomy	3	3.26
Laparoscopic procedure	No	59	64.13
	Yes	33	35.87
Stage according to AJCC 8th edition	1	27	29.35
	2	32	34.78
	3	22	23.91
	4	11	11.96
Positive family history of colorectal cancer	No	86	93.48
	Yes	6	6.52

M—mean; SD—standard deviation; Me—median.

**Table 7:** IRS classification.

**Percentage of Positive Cells**	**X**	**Intensity of Staining**
0—No visible cells with expression 1—<10% 2—10–50% 3—51–80% 4—>80%		0—No visible reaction 1—Weak staining 2—Mild staining 3—Intense staining
**IRS Classification**		
0–1 pts.—no expression 2–3 pts.—weak expression 4–8 pts.—mild expression 9–12 pts.—strong expression		

## Data Availability

The data presented in this study are available on request from the corresponding author. The data are not publicly available due to privacy issues.
